# Supplementary material for: Trp Fluorescence Redshift during HDL Apolipoprotein Denaturation Is Increased in Patients with Coronary Syndrome in Acute Phase: A New Assay to Evaluate HDL Stability
Source: Int J Mol Sci. 2021 Jul 22;22(15):7819. doi: 10.3390/ijms22157819 (PMC8345965; doi:10.3390/ijms22157819)
Supplement: Supplementary file 1 [file ijms-22-07819-s001.zip › ijms-1274828-SI.pdf]

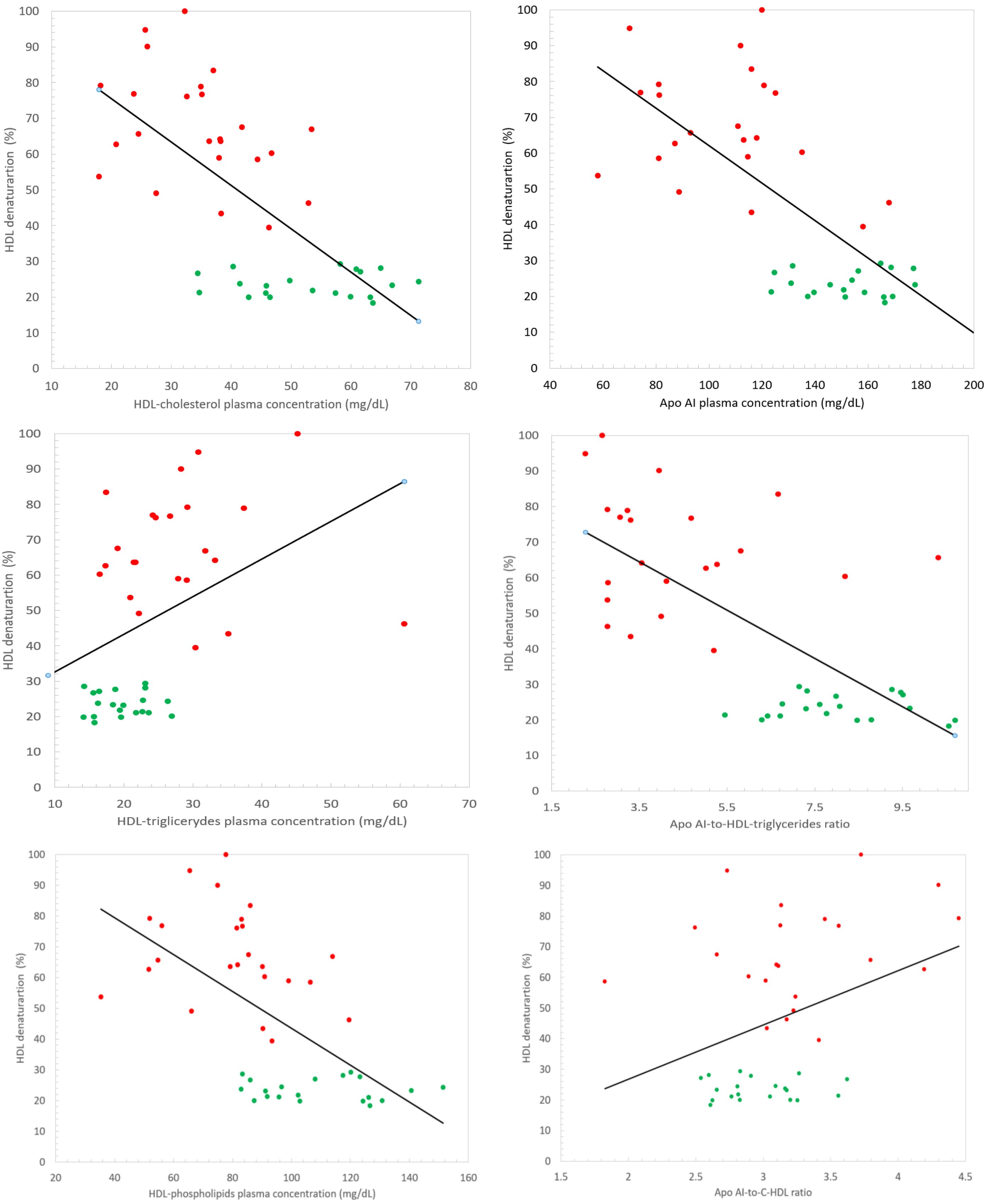

Figure S1. Plots and correlations trends of the components of HDL and the percentage of HDL denaturation. Data patients were represented in red and controls in green. Lines represent the regression trends. Only the plots of with significant correlations in Table 2 are shown.
